# Supplementary figures and images for: Simulation of the COVID-19 epidemic on the social network of Slovenia: Estimating the intrinsic forecast uncertainty
Source: PLoS One. 2020 Aug 27;15(8):e0238090. doi: 10.1371/journal.pone.0238090 (PMC7451520; doi:10.1371/journal.pone.0238090)

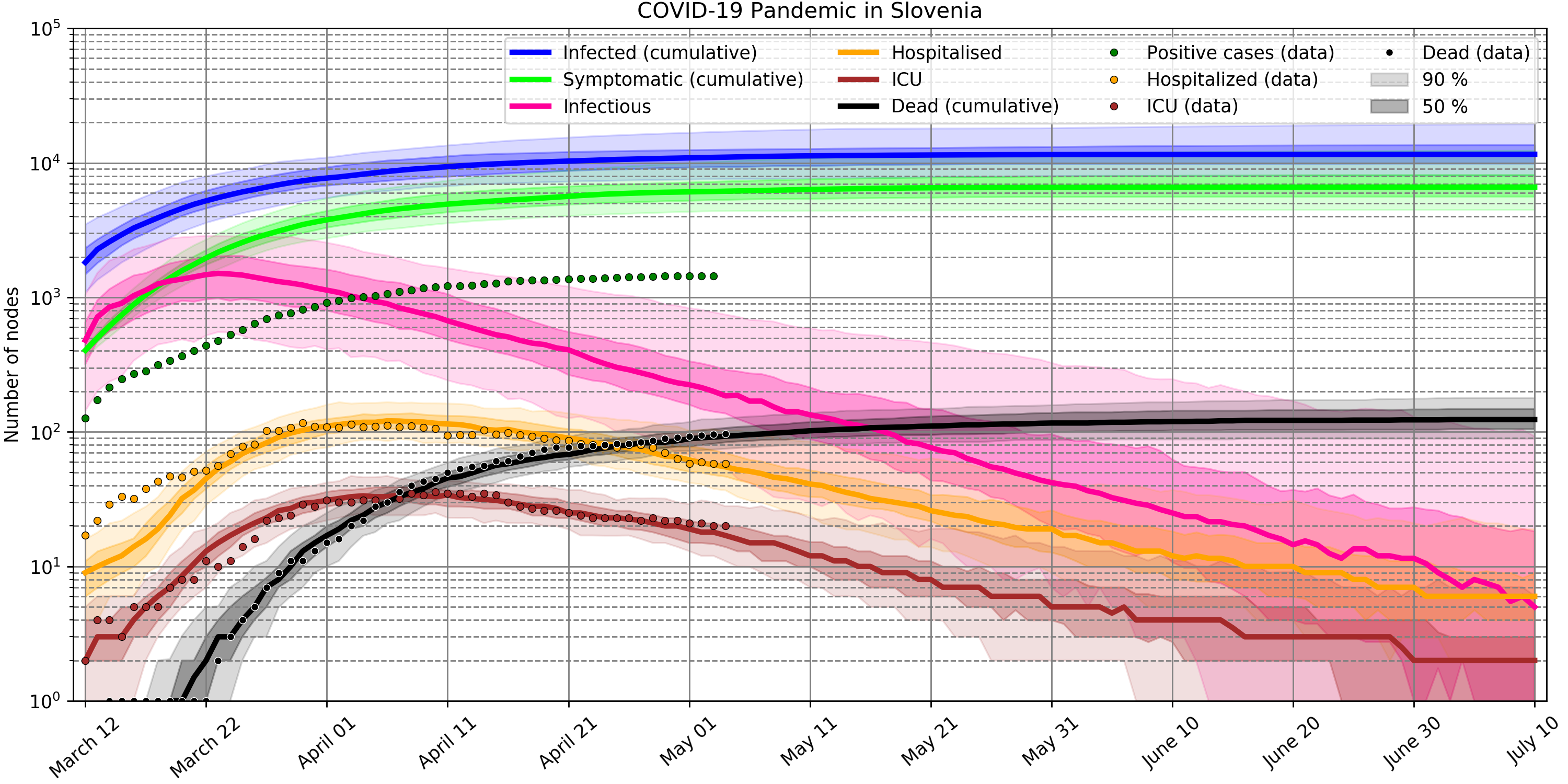

Supplement: S1 File — The core program korona_final.py is written in Python 2.7 and requires standard scipy, numpy and matplotlib. The most time-consuming procedures of the software are written in Fortran 90. Python binding are created using F2PY [94]: f2py -c generate_connections.f90 -m generate_connections. (ZIP) [file pone.0238090.s001.zip › data/data_Fig6/potek_pandemije_2020_05_05.png]
